# Defining the landscape of patient harm after osteopathic manipulative treatment: synthesis of an adverse event model

**DOI:** 10.1186/s12906-023-04230-2

**Published:** 2023-11-13

**Authors:** Mark D. Unger, Jackilyn N. Barr, Jacob A. Brower, Joseph C. Kingston, Gregory R. Heller, Joy L. Palmer

**Affiliations:** 1 Osteopathic Neuromusculoskeletal Medicine Residency, Graduate Medical Education Services, Liberty University College of Osteopathic Medicine, 2321 Wards Road, Lynchburg, VA 24502 USA; 2 Department of Osteopathic Manipulative Medicine and Osteopathic Principles and Practices, Liberty University College of Osteopathic Medicine, 306 Liberty View Lane, Lynchburg, VA 24502 USA

**Keywords:** Osteopathic manipulative treatment, Osteopathic manipulative medicine, Neuromusculoskeletal medicine, Adverse event outcome, Adverse event, Patient safety, United States, Outpatient, Quality, Pain

## Abstract

**Background:**

In the United States, osteopathic manipulative treatment (OMT), is a popular complementary physical health approach for the treatment of neuromusculoskeletal disorders. However, post-OMT adverse events (AEs) are poorly defined in terms of frequency, severity, and temporal evolution. To date, no benchmark for patient safety exists. To improve understanding in this field, we set out to model the landscape of patient harm after OMT.

**Methods:**

We conducted a comprehensive search of all available primary clinical research studies reporting on the occurrence of post-OMT AEs in nonpregnant, adult outpatients treated by an osteopathic physician in the United States. The methodology of eligible studies was then reviewed to select those containing the minimum required dataset to model the post-OMT AEs. The minimum required dataset consisted of four model parameters: ‘post-OMT interval’, ‘OMT encounters with post-OMT interval assessment’, ‘AEs preceded by an OMT encounter’, and ‘AE severity.’ We used the dataset extracted from selected studies to calculate a patient safety benchmark defined as the incidence rate of AEs per 100 post-OMT interval-days.

**Results:**

From 212 manuscripts that we identified, 118 primary clinical research studies were assessed for eligibility. A total of 23 studies met inclusion criteria for methodological review, of which 13 studies passed and were selected for modeling. Mild AEs were the most frequent, accounting for *n* = 161/165 (98%) of total AEs observed in the literature. The cumulative incidence of mild AEs was also significantly greater (*P* = 0.01) than both moderate and severe grades. The benchmark incidence rate was 1.0 AEs per 100 post-OMT interval-days.

**Conclusions:**

The majority of post-OMT AEs observed in the primary clinical literature were of mild severity. Modeling of the combined dataset on post-OMT AEs allowed for the derivation of a patient safety benchmark that, to date, has not been established in the field of osteopathic manipulative medicine. Additional research is needed to improve model resolution during the post-OMT period. This work conceptualized a model for identifying and grading post-OMT AEs, which should facilitate future comparisons between institutions in order to continually improve patient safety standards in the field of osteopathic manipulative medicine.

## Background

Osteopathic manipulative treatment (OMT) is a complementary physical health approach that ranks fourth among the most popular options used by adults in the United States [1, 2]. OMT comprises a group of manual techniques performed by an osteopathic physician where manual forces are applied in a therapeutic fashion to improve physiologic function and support homeostasis that has been altered by somatic dysfunction (SD) [3]. Somatic dysfunction is the impaired or altered function of related skeletal, arthrodial, myofascial, vascular, lymphatic, and neural structures [3]. SD is characterized by clinical signs of positional asymmetry, restricted range of motion, tissue texture abnormalities, and tenderness [3]. An osteopathic physician assesses SD during the osteopathic structural exam (OSE), which guides the administration of OMT to restore body function [3]. A recent overview of systematic reviews and meta-analyses of randomized controlled trials (RCTs) studying OMT for any condition concluded that the available evidence may support the effectiveness of OMT in adults with musculoskeletal disorders [4]. On the other hand, the safety of OMT is less clearly defined as many clinical studies did not report the occurrence of adverse events (AEs) after OMT [4].

In an editorial, one prominent osteopathic physician proposed that the current lack of sufficient post-OMT safety data is due to the rarity of severe AEs, which limits the feasibility of conducting much needed clinical trials that are appropriately powered [5]. While severe AEs after OMT are rare, underestimation may be unlikely because severe AEs are reportable occurrences [5, 6], clinically profound, and characterized by uncommon, debilitating symptoms and overt physical signs [7]. Despite being more common than severe AEs, mild AEs are more likely to be underestimated because the associated symptoms involve a transient or familiar patient experience with subtle or absent clinical signs [7–12]. Mild AEs often consist of pain, the patient-specific symptom defined as an unpleasant sensory and emotional experience associated with, or resembling that associated with, actual or potential tissue damage [7, 13, 14].

Underestimation of mild AEs is further enhanced in the outpatient setting where the majority of OMT is administered [6, 15]. Here, the reliability of self-reported outcomes is undermined by the patient’s ability to recall if and when an undesirable symptom occurred during the post-OMT interval of days, weeks, or months leading up to the scheduled follow-up appointment. Recall bias equally confounds characterization of symptom quality and localization by the patient. This situation limits a physician’s ability to judge whether the undesirable symptom constitutes an AE – any unfavorable or unintended disease, sign, or symptom (including an abnormal laboratory finding) that is temporally associated with the use of a medical treatment or procedure, and that may or may not be considered related to the medical treatment or procedure – and if that condition, being causally related to the medical treatment or procedure, may be classified as an adverse event outcome (AEO) [16].

Therefore, we recognized three barriers to progress in understanding the safety of OMT. One: while the majority share of post-OMT AEs is assumed to be mild or moderate, analysis of outcomes for this distinct therapeutic class has been diluted by the prevailing non-osteopathic manual therapy literature. Two: osteopathic physicians lack a common method to identify and grade post-OMT AEs that retains clinical utility for the assessment of pain and other forms of suffering marked by significant interindividual variability. Three: current models for conceptualizing the safety of OMT are inadequate because, to date, no benchmark has been established to compare patient outcomes between clinical institutions.

To overcome these barriers, we performed a comprehensive search of the available primary clinical literature reporting on adverse patient outcomes after OMT in nonpregnant, adult outpatients treated by an osteopathic physician in the United States. We adopted a set of definitions to identify and grade post-OMT AEs. Next, we devised a set of four parameters – ‘post-OMT interval’, ‘OMT encounters with post-OMT interval assessment’, ‘AEs preceded by an OMT encounter’, and ‘AE severity’ – to model post-OMT AEs. We used data from eligible studies that passed methodological review to populate model parameters. The cumulative incidence of post-OMT AEs was calculated and modeled to derive a novel patient safety benchmark in the field of osteopathic manipulative medicine, namely the incidence rate of post-OMT AEs.

## Methods

### Search strategy

A comprehensive literature search was performed as described below with the most recent search date being May 14, 2023. The authors used the available institutional medical database subscriptions, including Ovid, Clinical Key, ProQuest, LWW Health Library, Medline Ultimate, PubMed, PubMedCentral, SAGE Journals, ScienceDirect, Scopus, Springer Link, EBSCO, Oxford Academic Journals, Nature, Taylor and Francis, Wiley Online Library, and Journal of the American Medical Association, and the scholarly literature search engine Google Scholar to obtain manuscripts that were restricted behind journal paywalls. As manuscripts were identified, their respective reference lists were tracked backward in time to identify relevant manuscripts. The following unfiltered search term was entered into the PubMed database: ("adverse event" OR "adverse effect" OR "adverse events" OR "adverse effects") AND osteopathic AND (OMT OR OMM). The manuscripts resulting from this search comprised level 1 of the search strategy. Manuscripts were categorized as either primary clinical research (prospective and retrospective studies), secondary clinical research (reviews, meta-analyses, and editorials), or out of scope (medical education manuscripts, clinical practice guidelines, and theses/abstracts). The titles and abstracts of references cited by each level 1 secondary clinical research manuscript were screened to identify additional primary and secondary clinical research manuscripts that appeared to be pertinent. These manuscripts comprised level 2 of the search strategy. Level 2 manuscripts were sorted as described for level 1 and so on, eventually producing levels 3, 4, and 5. In other words, the search strategy required tracking reference lists backward in time through a chain of five referenced manuscripts. Duplicate references, identified by title, author list, and year, were discarded to avoid duplication. An attempt was made to obtain a copy of all manuscripts using the search tools described above. The authors did not contact the corresponding authors to obtain inaccessible manuscripts and, because the authors did not pay for manuscript access as the enclosed study was not funded, manuscripts that could not be accessed by the authors were labelled as ‘unable to obtain’ and were not assessed.

### Eligibility criteria

All primary clinical research manuscripts that were identified through the search strategy were assessed to determine eligibility for inclusion in the subsequent methodological review. Inclusion criteria were as follows: study subjects age ≥ 18 years old, study subjects nonpregnant or ≥ 1 year postpartum, study subjects received OMT, OMT performed or supervised by an osteopathic physician, non-inpatient clinical setting (outpatient clinic, nursing home, emergency department), and study conducted in the United States. Manuscripts that failed to satisfy all six inclusion criteria were excluded.

### Methodological review

The methodologies of eligible studies were reviewed to select those containing the data required for model synthesis. We selected studies that reported data in terms of the following four parameters defined in greater detail in Table 1: ‘post-OMT interval’, ‘OMT encounters with post-OMT interval assessment’, ‘AEs preceded by an OMT encounter’, and ‘AE severity.’ Post-OMT AE count data was assessed on an encounter-specific basis: OMT encounters involving the administration of OMT alone were counted while encounters involving the co-administration of OMT and another intervention were excluded. Because all eligible prospective, interventional studies that contributed data for model development implemented the use of predefined study protocols, post-intervention monitoring, and a maximum post-OMT interval of nine days, encounters were included whether or not the subjects had previously received OMT outside of study enrollment. Studies that did not describe the methods used in sufficient detail to inform the value of all four parameters failed methodological review and were excluded.

**Table 1:** Terms and definitions used to conceptualize the adverse event model

Term	Definition^a^
Patient harm	• A distinct occurrence involving temporary or permanent impairment of the physical, emotional, or psychological function or structure of the body and/or any undesired or deleterious effect arising therefrom [17] • Clinically manifest as subjective or objective patient data • Examples: suffering, injury, disability, disease, death [17] • Types: AE, AEO
Suffering	• The experience of anything subjectively unpleasant [17] • Examples: pain, malaise, nausea, depression, agitation, alarm, fear, grief [17]
AE	• A type of patient harm occurring after a treatment or procedure that may or may not be caused by that treatment or procedure [7, 16, 17] • Classified as new or worsening relative to the patient’s experience and history: o New: peak NRS score ≥ 2 points [18] o Worsening: peak NRS score ≥ 2 points higher than prior baseline NRS score [18] • Includes all AEOs as a subset
AEO	• A condition or event that is attributed to the adverse event and is the result or conclusion of the adverse event [16]
NRS	• An 11-point numeric scale used to measure the quantity of harm, where selection of the number 0 indicates the absence of harm and selection of the number 10 indicates maximum possible harm [18] • Used to identify the type of patient harm that occurred [19] • Does not indicate the severity of patient harm • Does not imply causality between an occurrence of patient harm and a prior treatment or procedure
AE severity	• Mild: CTCAE Grade 1, defined as mild pain, asymptomatic or mild symptoms; clinical or diagnostic observations only; intervention not indicated [7] • Moderate: CTCAE Grade 2, defined as moderate pain, minimal, local, or noninvasive intervention indicated; limiting age-appropriate instrumental ADL [7] • Severe: CTCAE Grade 3, defined as severe pain, medically significant but not immediately life-threatening; hospitalization or prolongation of hospitalization indicated; disabling; limiting self-care ADL [7] • Life-threatening: CTCAE Grade 4, defined as life-threatening consequences with urgent intervention indicated [7] • Death: CTCAE Grade 5, defined as the occurrence of death [7] • For data modeled in the enclosed manuscript, the CTCAE SOC “Musculoskeletal and Connective Tissue Disorders” was used to grade all OMT encounters. For a patient harm occurrence that would be better categorized as a non-musculoskeletal or non-connective tissue disorder, the affected body region would be matched to the corresponding SOC prior to using the subjective and objective patient data to assign the best matched CTCAE Grade as defined under the corresponding SOC • Used to grade AEs and AEOs • Not used to identify the type of patient harm that occurred • Does not imply causality between an occurrence of patient harm and a prior treatment or procedure
ADL	• Instrumental ADL: refers to preparing meals, shopping for groceries or clothes, using the telephone, managing money, etc. [7]
Self-care ADL	• Self-care ADL: refers to bathing, dressing, undressing, feeding self, using the toilet, taking medications, and not bedridden [7]
SOC	• The highest level of hierarchy for identification by anatomical or physiological system, etiology, or purpose [7] • Within each SOC, patient harm occurrences are listed and accompanied by descriptions of severity [7]
OMT encounter	• A clinical appointment during which the procedure of OMT is administered by an osteopathic physician to an informed and consenting patient [3]
Post-OMT interval	• The period of time beginning immediately at the conclusion of an OMT encounter and ending when the patient completes a post-OMT interval assessment either before receiving the next scheduled OMT procedure or at the conclusion of study participation
Post-OMT interval assessment	• A patient interview where the purpose is to specifically assess for any occurrence of patient harm after a preceding OMT encounter • For each occurrence of patient harm, the interviewer seeks to characterize the harm according to its identity, grade, and temporal evolution • The date of the prior OMT encounter is time point zero and the date of post-OMT interval assessment marks the end of the post-OMT interval
OMT encounter with post-OMT interval assessment	• Any OMT encounter for which the patient subsequently receives a post-OMT interval assessment
AEs preceded by an OMT encounter	• Any AE occurring after an OMT encounter
Cumulative incidence of post-OMT AEs	• The number of ‘AEs preceded by an OMT encounter’ divided by the number of ‘OMT encounters with post-OMT interval assessment’ multiplied by 100% • Adapted from the generic definition of cumulative incidence [20, 21]
Post-OMT AE incidence rate	• The number of ‘AEs preceded by an OMT encounter’ divided by the number of ‘post-OMT interval-days.’ • Adapted from the generic definition of incidence rate [20, 21]
Post-OMT interval-days	• The total number of ‘post-OMT interval’ days across all modeled studies
Benchmark	• A measure of comparative performance [17] • A point of reference or standard by which something can be measured, compared, or judged [17] • Defined here as the incidence rate ‘AEs per 100 post-OMT interval-days.’

Legend: ^a^Bullets are used to separate the components of each definition by source. Bullets containing an in-line citation indicate the source is referenced in the manuscript. Bullets lacking an in-line citation indicate the term and/or definition is undefined or not standardized in the literature and therefore originated in the context of the enclosed manuscript. *ADL* Activities of daily living, *AE* Adverse event, *AEO* Adverse event outcome, *CTCAE* Common Terminology Criteria for Adverse Events, *CS* Counterstrain, *MFR* Myofascial release, *NRS* Numeric rating scale, *OMT* Osteopathic manipulative treatment, *SOC* System organ class

### Adopted terms and definitions

Various terms and definitions were selected to standardize the identification and grading of post-OMT AEs observed in the primary clinical literature. We adopted existing terms and definitions that were previously established in the literature. We formulated original terms and definitions as needed if no prior resource provided context. Table 1 lists the adopted terms and definitions.

### Data extraction, model development, and statistics

Studies that passed the methodological review contributed data for model synthesis. In addition to populating model parameters, study characteristics were recorded to support interpretation of results. Extraction of the parameter ‘AE severity’ was standardized as follows. If a study used the patient’s own words (POW) to indicate the occurrence of an AE, the Common Terminology Criteria for Adverse Events (CTCAE) grading scale was used to assign AE severity based on the CTCAE term and grade that best matched the affected anatomical area and POW. If a study reported the severity of AEs using an unreferenced grading system, a CTCAE grade was assigned by translating the reported severity levels in terms of the best matched CTCAE grades. Severity data was adopted as reported for studies that used the CTCAE grading system by reference. All AEs reported in the literature were categorized as AEs whether or not a causal relationship between the adverse patient outcome and preceding OMT encounter was declared and whether or not the authors declared what criteria were used to identify each AE. The parameter ‘post-OMT interval’ was converted into days for all studies. Studies with a post-OMT interval of less than 24 h were included under the post-OMT interval of one day. The OMT protocol for each study was used to construct the assessment timeline for all encounters prior to extracting the parameters ‘OMT encounters with post-OMT interval assessment’ and ‘AEs preceded by an OMT encounter.’ To calculate cumulative incidence of AEs, the parameter ‘AEs preceded by an OMT encounter’ was divided by the parameter ‘OMT encounters with interval assessment’ and the resulting decimal was multiplied by 100%. The model was graphed using JMP 15.2.1, SAS Institute Inc., Cary, NC and Inkscape 1.2.2, The Inkscape Project, www.inkscape.org. Using JMP, we conducted a one-way ANOVA with blocking by study identity followed by Tukey’s post-hoc test for multiple comparisons to assess for significant differences in cumulative incidence between AE severity grades. The cutoff for statistical significance was set at *P* < 0.05. To calculate the benchmark incidence rate per 100 post-OMT interval-days, total modeled ‘AEs preceded by an OMT encounter’ was divided by total modeled post-OMT interval-days and multiplied by 100. Total modeled post-OMT interval-days was calculated by adding together the post-OMT interval days across all modeled OMT encounters.

## Results

### Search results and manuscript screening

**Fig. 1:**
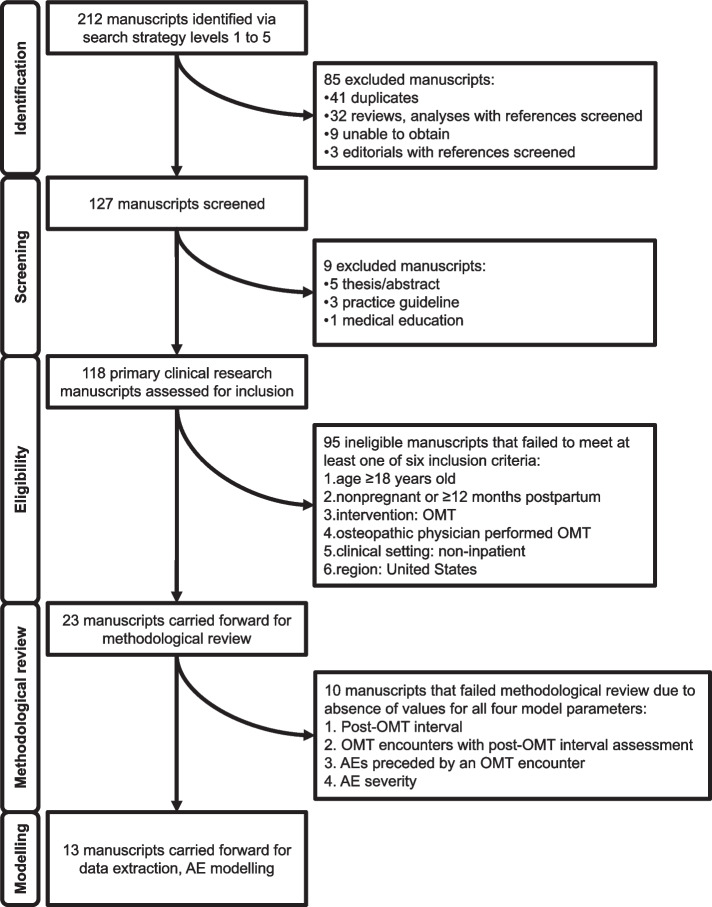
Flow diagram of manuscripts identified, screened, methodologically reviewed, and included in subsequent AE modeling. AE-Adverse event; OMT-Osteopathic manipulative medicine

As depicted in Fig. 1, a total of 212 manuscripts were identified through the five-level search strategy. After reference backtracking of all primary and secondary clinical research manuscripts, 85 manuscripts were excluded. This resulted in 127 manuscripts that were screened to identify a total of 118 primary clinical research studies. The remaining 9 manuscripts were not primary clinical research and were excluded.

### Eligibility of primary clinical research studies

As depicted in Fig. 1, 95 primary clinical research studies were assessed to be ineligible for subsequent methodological review due to a failure of each study to meet all six inclusion criteria. A total of 23 studies met all six inclusion criteria and were carried forward for methodological review.

### Methodological review of eligible studies and study characterization

We reviewed 23 eligible studies to determine if the respective methodologies supported data interpretation at the encounter-specific level. A total of 10 studies failed methodological review. The remaining 13 studies passed methodological review. Table 2 shows the characteristics of the passing studies which included a total of n = 1,237 patients. Females comprised approximately 67.0 ± 20% (mean ± SD) of the population across all studies. The approximate age of all study subjects was 55 ± 15 years (mean ± SD). The majority of studies, 9/13 (69%), conducted an OSE to diagnose SD and guide the administration of OMT. For studies that reported the duration of OMT techniques performed during each OMT encounter, the average duration of OMT interventions was 21 ± 12 min (mean ± SD). The selection of specific OMT techniques was variable across the studies. A combination of direct, indirect, active, and passive OMT techniques were administered. The occurrence of AEs was reported in terms of POW for the majority of studies, 10/13 (77%). For the remaining studies, AEs were reported in terms of clinical signs, 1/13 (8%) or AEs were not detected, 2/13 (15%).

**Table 2:** Characteristics of studies that passed methodological review

Study reference number	Study design^a^	Patients in OMT group (n)	Mean OMT encounters per patient	Female^b^ (% of all subjects)	Age^b^ (mean yr ± sd)	Indication for OMT	Duration of OMT encounter and techniques performed^c^	Approach to AE/AEO grading
[22]	Pilot	10	1	70.0	45.0 ± 15.0	• Headache ≥ 3 months after mild TBI	• 4 techniques/encounter • Non-protocolized: ME, MFR, CS, Suboccipital release	• No AEs/AEOs detected during post-OMT interval assessments
[6]	Observational	884	2	76.0	51.8 ± 15.8	• SD	• “Real world” office visits • Non-protocolized: HVLA, CS, FPR, ME, ART, Still, MFR, ST, Visceral, OCMM, Indirect, Functional, BLT, LAS	• 5-point scale • POW • Retrospective chart review to grade AEs: mild, moderate, serious
[23]	RCT	29	1	62.0	29.0 ± 8.0	• Musculoskeletal neck pain < 3 wk • SD	• Up to 5 min/encounter • Non-protocolized: HVLA, ME, ST	• POW
[24]	Pilot	10	1	100	47.0 ± 10.0	• Chronic asthma	• 10–15 min/encounter • Protocolized: BLT, Still, Direct, MFR	• POW
[11]	RCT	18	5	44.0	68.0 ± 8.0	• COPD	• 5–10 min/encounter • Protocolized: Lymphatic pump, Rib raising, MFR	• POW
[9]	RCT	17	1	56.0	69.6 ± 6.6	• COPD • SD	• 20 min/encounter • Non-protocolized + Protocolized: MFR, HVLA, ME, ST, Rib raising, Suboccipital decompression, Lymphatic pump	• POW
[25]	RCT	9	12	20.0	72 ± 11.3	• Motor function, balance in PD	• 30 min/encounter • Protocolized: Park-OMM protocol	• POW
[10]	RCT	7	9	86.0	82.3 ± 4.7	• Influenza vaccine recipients • SD	• 15 min/encounter • Non-protocolized + Protocolized: ME, CS, MFR, Direct, ART, Paraspinal inhibition, Rib raising, Lymphatic pump, Splenic pump	• POW
[26]	Feasibility	6	3	73.1	52.5 ± 11.8	• Peripheral vertigo > 3 mo • SD	• 45 min/encounter • Non-protocolized: CS, MFR, BLT, ST, HVLA, ART	• No AEs/AEOs detected during post-OMT interval assessments
[19]	RCT	27	4	76.0	42.1 ± 13.5	• Neck pain > 3 mo • SD	• 30 min/encounter • Protocolized: HVLA, ST, ME, MFR, ART	• POW • 2-point NRS increase = AE • CTCAE grades
[27]	Feasibility	11	6	82.0	50.5 ± NR	• Pain in FM • SD	• 30 min/encounter • Non-protocolized: MFR, ME, CS, FPR, LAS, HVLA, OCMM	• Survey items • POW • Side effect severity: mild, moderate, severe
[28]	Pilot	18	4	62.5	64.5 ± NR	• Peripheral vertigo > 3 mo • SD	• 4 techniques/encounter • Non-protocolized: ME, CS, MFR, BLT	• Survey items • POW • Mild, moderate, severe
[29]	RCT	191	6	63.0	41.0, 29–51 (median, IQR)	• Low back pain ≥ 3 mo • SD	• 15 min/encounter • Non-protocolized: HVLA, ART, ST, MFR, ME	• Description of clinical signs

Legend: ^a^All studies that passed methodological review were found to be prospective and interventional with the exception of [6] which was observational. Additional design traits are listed as reported. ^b^Gender and age distributions were calculated for patients enrolled in the OMT group for all studies except [27] where gender and age data were reported for all patients regardless of treatment group. ^c^The OMT duration and techniques represent the OMT intervention described in the methods section of each study. *AE* Adverse event, *AEO* Adverse event outcome, *ART* Articulatory, *BLT* Balanced ligamentous tension, *COPD* Chronic obstructive pulmonary disease, *CS* Counterstrain, *CTCAE* Common Terminology Criteria for Adverse Events, *FM* Fibromyalgia, *FPR* Facilitated positional release, *HVLA* High-velocity, low amplitude, *LAS* Ligamentous articular strain, *ME* Muscle energy, *MFR* Myofascial release, *NR* Not reported, *NRS* Numerical rating scale, *OCMM* Osteopathic cranial manipulative medicine, *OMM* Osteopathic manipulative medicine, *OMT* Osteopathic manipulative treatment, *OSE* Osteopathic structural exam, *PD* Parkinson’s disease, *POW* Patient’s own words, *RCT* Randomized controlled trial, *SD* Somatic dysfunction, *ST* Soft tissue, *TBI* Traumatic brain injury

### Synthesis of the post-OMT AE model

**Fig. 2:**
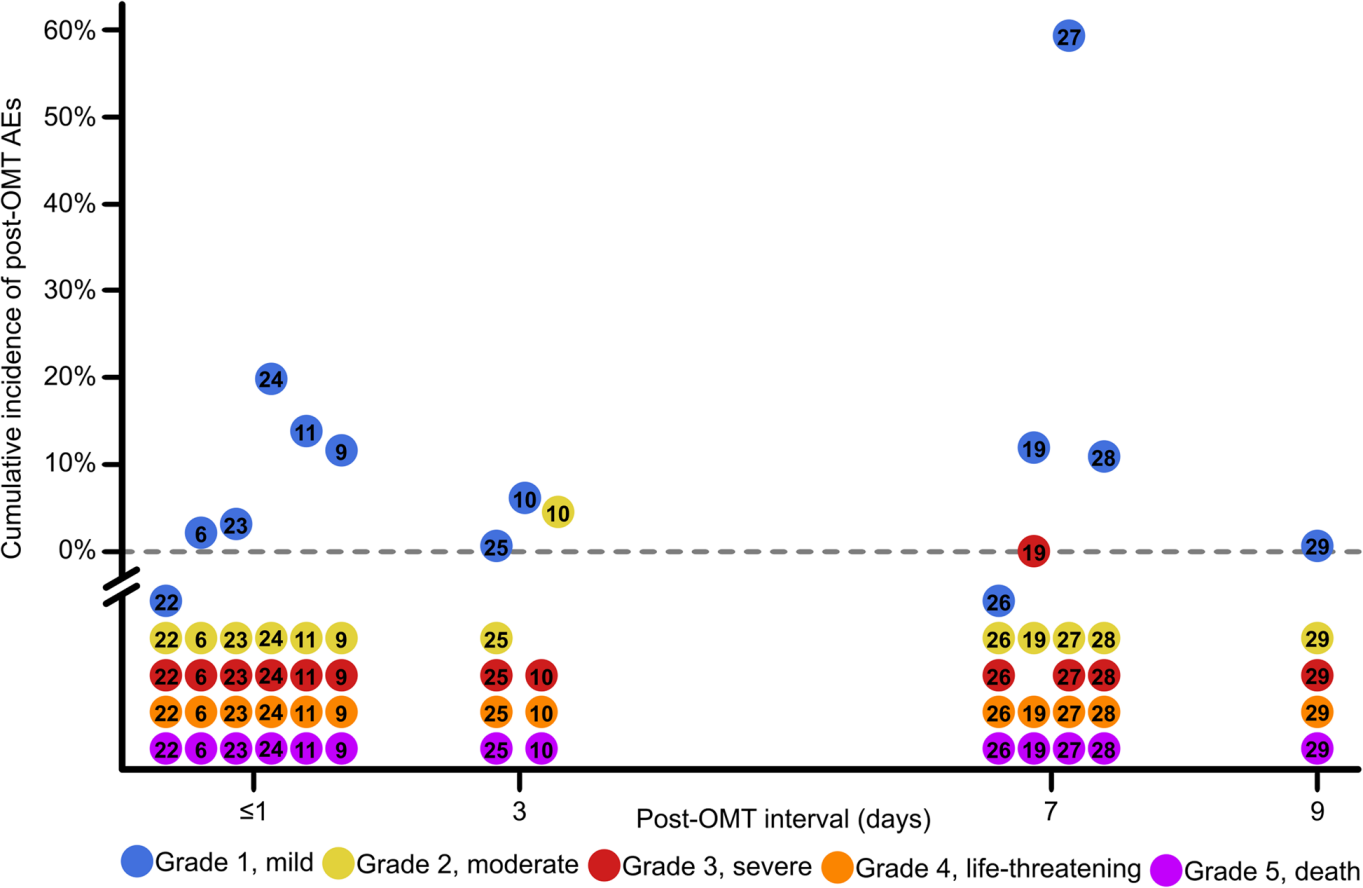
Visualizing the landscape of post-OMT harm. Legend: Cumulative incidence of post-OMT AEs is plotted against the post-OMT interval day upon which the patient was assessed to determine if any undesired symptoms or AEs since the preceding OMT encounter had occurred. The post-OMT interval (x-axis, days) ranged from ≤ 1 to 9 days and represents the day of patient assessment. Cumulative incidence of post-OMT AEs (y-axis, %) represents the number of AEs preceded by an OMT encounter divided by the number of OMT encounters with post-OMT interval assessment, multiplied by 100%. A total of 13 eligible studies passed methodological review and therefore contributed data for modeling. For reference, Table 3 tabulates the number of AEs preceded by an OMT encounter per AE severity grade and the number of OMT encounters with post-OMT interval assessment per study. Study identity is indicated by the manuscript reference number on each data point. Readers are directed to each numbered reference for study-specific descriptions of the observed AEs. AE severity is indicated by data point color: blue = mild (CTCAE Grade 1), yellow = moderate (Grade 2), red = severe (Grade 3), orange = life-threatening (Grade 4), and purple = death (Grade 5). A double slash breaks the y-axis at 0% cumulative incidence (horizontal dotted line) to indicate that all points clearly below the dotted line correspond to a value of 0% cumulative incidence. AE-Adverse event; CTCAE-Common Terminology Criteria for Adverse Events; OMT-Osteopathic manipulative medicine

Table 3 lists the data extracted from each study that passed methodological review. Across 13 studies, reports of mild AEs, *n* = 161/165 (98%), of total AEs outnumbered both moderate, *n* = 3/165 (2%) of total AEs, and severe, *n* = 1/165 (1%), of total AEs. No life-threatening AEs or patient deaths were observed. Figure 2 depicts the cumulative incidence of post-OMT AEs by AE severity grade versus post-OMT interval for each study. After determining that the independent effect of study identity was not significant (*P* = 0.49), the cumulative incidence of mild AEs was found to be significantly greater (*P* = 0.01) than both moderate and severe AEs while the difference between moderate and severe AEs was not significant. Data for post-OMT interval days 2, 4, 5, 6, and 8 were not observed in the modeled studies. Across all severity grades and studies, *n* = 165 AEs were observed after *n* = 3,778 OMT encounters (approximately 5%). Therefore, the benchmark incidence rate of post-OMT AEs per 100 post-OMT interval-days was [165 AEs]/[16,014 post-OMT interval-days]x[100] = 1.0 AEs per 100 post-OMT interval-days.

**Table 3:** Model parameters extracted from studies that passed methodological review

Study reference number	AE severity^a^	AEs preceded by an OMT encounter	OMT encounters with post-OMT interval assessment	Post-OMT interval (days)	Cumulative incidence of post-OMT AEs^b^	Post-OMT interval-days^c^
[22]	Mild	0	10	1	0.0	10
	Moderate	0				
	Severe	0				
[6]	Mild	45	1847	1	2.4	1847
	Moderate	0				
	Severe	0				
[23]	Mild	1	29	1	3.4	29
	Moderate	0				
	Severe	0				
[24]	Mild	2	10	1	20	10
	Moderate	0				
	Severe	0				
[11]	Mild	13	93	1	14	93
	Moderate	0				
	Severe	0				
[9]	Mild	2	17	1	11.8	17
	Moderate	0				
	Severe	0				
[25]	Mild	1	109	3	0.9	327
	Moderate	0				
	Severe	0				
[10]	Mild	4	63	3	11.1	189
	Moderate	3				
	Severe	0				
[26]	Mild	0	18	7	0.0	126
	Moderate	0				
	Severe	0				
[19]	Mild	36	298	7	12	2086
	Moderate	0				
	Severe	1				
[27]	Mild	39	66	7	59	462
	Moderate	0				
	Severe	0				
[28]	Mild	8	72	7	11.1	504
	Moderate	0				
	Severe	0				
[29]	Mild	10	1146	9	0.9	10,314
	Moderate	0				
	Severe	0				
Variables, formulae		X	Y	Z	([total X]/Y)*100%	Y*Z

Legend: ^a^Each AE reported in the literature was classified as an AE whether or not a causal relationship between the adverse patient outcome and preceding OMT encounter was reported. AE severity grades “life-threatening” and “death” are omitted from the table as no AEs were observed in either category across all modeled studies. ^b^Cumulative incidence of post-OMT AEs for each study was calculated as follows: ([total X]/Y)*100%. Cumulative incidence for each study was calculated across all AEs regardless of AE severity. ^c^Post-OMT interval-days for each study was calculated as follows: Y*Z. *AE* Adverse event, *OMT* Osteopathic manipulative treatment

## Discussion

We synthesized a model to standardize the evaluation of adverse patient outcomes after OMT. To do so, we addressed three barriers to progress in the field. First, we performed a search of the existing primary clinical literature to identify studies involving the administration of OMT by osteopathic physicians in the United States. That decision was made because prior efforts to determine the rate of AEs after OMT have been dominated by the body of literature reporting patient outcomes after manual therapy performed by non-osteopathic healthcare professionals – massage therapists, chiropractors, physical therapists, and non-physician osteopaths – who possess different practice rights in the United States as compared to osteopathic physicians [3, 5, 30]. AEs after manual techniques performed by non-physicians have been reported for procedures similar to the osteopathic technique high-velocity, low-amplitude (HVLA) [5, 8, 30]. HVLA represents one of at least twelve unique types of OMT practiced by osteopathic physicians in the United States [3]. To illustrate the differences among manual techniques, one early review on the safety of manipulative treatment from 1925 to 1993 found no cases of injury after muscle energy (ME), indirect, and fascial OMT [8]. The majority of severe AEs, approximately 14% of which resulted in fatal cerebrovascular accidents, occurred after cervical HVLA performed in extension [8]. Osteopathic physicians in the United States are trained to administer cervical HVLA in a neutral or flexed position due to the aforementioned negative outcomes, thereby establishing a fundamental difference in the procedure of cervical HVLA as performed by osteopathic physicians relative to non-osteopathic healthcare professionals [28, 31–35]. This may explain the relative difference in overall cumulative incidence observed after OMT, approximately 5% for AEs, versus that observed after manual therapy, approximately 22% for AEs alone [36]. However, the difference between HVLA administered by an osteopathic physician as compared to HVLA-type techniques administered by others may be less significant in light of a more recent systematic review that found a small association between chiropractic neck manipulation and cervical artery dissection [37]. That review found the quality of evidence to be very low [37].

Second, we found that no common method has been used to identify and grade post-OMT AEs. We viewed this problem from the osteopathic patient’s perspective, commonly one who seeks treatment for a chronically painful musculoskeletal disorder [15]. Two such patients, for example, both diagnosed with mechanical low back pain, do not suffer the same discomfort and disability because the experience of pain is highly variable between individuals [13, 14]. However at the same time, each patient’s pain experience is restricted to their own sensorium which may explain why various chronic pain populations demonstrate similar thresholds for what constitutes a clinically important difference in symptom progression [18, 38]. Our search found one study that applied this concept in the setting of post-OMT safety to identify AEs and defined an increase of two numeric rating scale (NRS) points from baseline to be the threshold for classifying a symptom as an AE and, further, applied the CTCAE grading scale to determine AE severity [19]. This approach stands out as the most rigorous of all studies included in our analysis. Most studies reported AEs in terms of POW and did not indicate a formal procedure for grading severity. As the authors of the more rigorous approach noted, counting all unfavorable symptoms as AEs regardless of change in NRS score from baseline would have inflated the incidence of AEs [19]. To build on their method, we proposed that a two-point increase in the NRS for any undesired symptom, new or worsening, should indicate the occurrence of an AE but not necessarily an AEO. This approach should increase model sensitivity for detecting AEs without negatively impacting specificity for those AEs that are judged to be AEOs.

Third, we report a patient safety benchmark against which future trials and quality improvement studies in the field of osteopathic manipulative medicine (OMM) may be compared. The metric – AEs per 100 post-OMT interval-days – is the incidence rate modeled from the combined dataset on post-OMT AEs that we extracted after assessment of the primary clinical literature. To the best of our knowledge, the incidence rate of post-OMT AEs has never been reported. The reason may be because all prior studies conducted one post-OMT interval assessment during each post-OMT interval thereby precluding measurement of incidence rate. By modeling all studies with a standard time parameter – ‘post-OMT interval’ – we were able to derive the incidence rate of observing an AE as a function of time elapsed since prior OMT.

This study is not without limitations. First, the lack of data for some time points, specifically post-OMT interval days 2, 4, 5, 6, and 8 as shown in Fig. 2, and the uneven distribution of data over time limits interpretation of this study. Unfortunately, we did not identify any eligible studies that were designed to assess for post-OMT AEs during the missing time points and, furthermore, none of the modeled studies that reported AEs used the term AEO or provided long-term follow-up. This makes measurement of how many AEs persisted to become AEOs difficult. One benefit of selecting incidence rate to benchmark our model is that the denominator assumes a constant probability of AEs occurring during the study period [20, 21]. Clinicians choosing to use Fig. 2 as a reference for causality assessment may consider model reliability greatest during the first seven post-OMT days because the majority of data points are found over this period. Nevertheless, the model highlights where additional research is needed to improve resolution of the post-OMT harm landscape. A second limitation is that one [6] of the 13 modeled studies included data corresponding to post-OMT AEs after OMT was administered by *n* = 1 allopathic physician and *n* = 1 Canadian osteopath. While these two clinicians comprised a minority among the remaining *n* = 41 osteopathic physicians included in that study, the authors did not report on the number of encounters attributed to these two non-osteopathic clinicians. A third limitation is that the enclosed study is not a systematic review and was not registered with PRISMA. A fourth limitation, due to the strictness of parameters used to construct the model, is that studies reporting AEs without documentation of the corresponding post-OMT interval were excluded. A fifth limitation is that of patient recall bias due to the inclusion of studies that reported post-OMT AEs in terms of POW.

## Conclusions

During the first nine days after OMT, AEs were observed to be mild in the majority of cases. The incidence rate was benchmarked at 1.0 AEs per 100 post-OMT interval-days based on modeling data extracted from the primary clinical literature. Future research is needed to improve model resolution during the initial post-OMT period. This study should assist current research on the safety of OMT by facilitating the identification and grading of AEs after OMT.

## Data Availability

All data generated or analyzed during this study are included in this published article.

## Abbreviations

ADL: Activities of daily living
AE: Adverse event
AEO: Adverse event outcome
CTCAE: Common Terminology Criteria for Adverse Events
HVLA: High-velocity, low-amplitude
ME: Muscle energy
NRS: Numeric rating scale
OMM: Osteopathic manipulative medicine
OMT: Osteopathic manipulative treatment
OSE: Osteopathic structural exam
POW: Patient’s own words
RCT: Randomized controlled trial
SD: Somatic dysfunction
SOC: System organ class
